# Rumen and fecal microbiomes are related to diet and production traits in *Bos indicus* beef cattle

**DOI:** 10.3389/fmicb.2023.1282851

**Published:** 2023-12-15

**Authors:** Liliane Costa Conteville, Juliana Virginio da Silva, Bruno Gabriel Nascimento Andrade, Tainã Figueiredo Cardoso, Jennifer Jessica Bruscadin, Priscila Silva Neubern de Oliveira, Gerson Barreto Mourão, Luiz Lehmann Coutinho, Julio Cesar Pascale Palhares, Alexandre Berndt, Sergio Raposo de Medeiros, Luciana Correia de Almeida Regitano

**Affiliations:** ^1^Embrapa Southeast Livestock, São Carlos, Brazil; ^2^Department of Genetics and Evolution, Federal University of São Carlos (UFSCar), São Carlos, Brazil; ^3^Computer Science Department, Munster Technological University, Cork, Ireland; ^4^Department of Animal Science, Center for Functional Genomics, University of São Paulo/ESALQ, Piracicaba, Brazil

**Keywords:** agricultural by-products, *Bos indicus*, feed efficiency, methane production, residual feed intake, residual methane emission, shotgun metagenomic sequencing

## Abstract

**Background:**

Ruminants harbor a complex microbial community within their gastrointestinal tract, which plays major roles in their health and physiology. Brazil is one of the largest producers of beef in the world and more than 90% of the beef cattle herds are composed of pure and crossbred Nelore (*Bos indicus*). Despite its importance to the Brazilian economy and human feeding, few studies have characterized the Nelore microbiome. Therefore, using shotgun metagenomics, we investigated the impact of diet on the composition and functionality of the Nelore microbiome, and explored the associations between specific microbial taxa and their functionality with feed efficiency and methane emission.

**Results:**

The ruminal microbiome exhibited significantly higher microbial diversity, distinctive taxonomic profile and variations in microbial functionality compared to the fecal microbiome, highlighting the distinct contributions of the microbiomes of these environments. Animals subjected to different dietary treatments exhibited significant differences in their microbiomes’ archaeal diversity and in the abundance of 89 genera, as well as in the functions associated with the metabolism of components of each diet. Moreover, depending on the diet, feed-efficient animals and low methane emitters displayed higher microbial diversity in their fecal microbiome. Multiple genera were associated with an increase or decrease of the phenotypes. Upon analyzing the functions attributed to these taxa, we observed significant differences on the ruminal taxa associated with feed efficient and inefficient cattle. The ruminal taxa that characterized feed efficient cattle stood out for having significantly more functions related to carbohydrate metabolism, such as monosaccharides, di−/oligosaccharides and amino acids. The taxa associated with methane emission had functions associated with methanogenesis and the production of substrates that may influence methane production, such as hydrogen and formate.

**Conclusion:**

Our findings highlight the significant role of diet in shaping Nelore microbiomes and how its composition and functionality may affect production traits such as feed efficiency and methane emission. These insights provide valuable support for the implementation of novel feeding and biotechnological strategies.

## Graphical abstract

The Nelore picture was taken by Gisele Rosso in 2023 and it belongs to Embrapa Southeast Livestock Multimedia: Image bank.

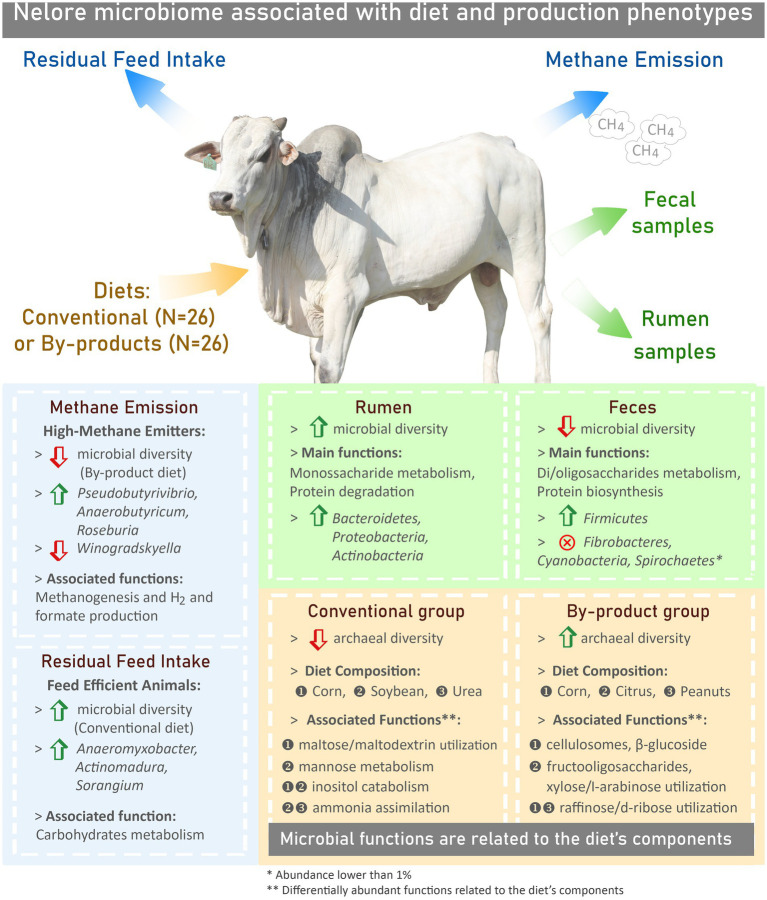

## 1 Introduction

Ruminants rely on the complex microbial community that resides in their gastrointestinal tract (GIT) to digest complex dietary components, develop and modulate the immune system, and protect against infections (Huws et al., 2018). Each segment of the GIT is a different, but not an independent environment, with distinct physiological and biochemical properties. This heterogeneity is also observed in their microbiome diversity (de Oliveira et al., 2013; Andrade et al., 2020). Beyond that, factors such as host age, diet, genetics, and interactions with the environment can also cause variations in microbiome composition and functionality (Henderson et al., 2015; Welch et al., 2021; Andrade et al., 2022; Martínez-Álvaro et al., 2022).

It is well known that diet has the greatest influence on the GIT microbiomes, with the potential to alter not only the microbial diversity as a whole but also the individual taxa abundance (Henderson et al., 2015; Andrade et al., 2022). Moreover, the ruminant’s efficiency in exploring their diet is mainly determined by the ability of the microbiome to convert the ingested feed and the microbial mass produced into metabolically available nutrients and energy that can be utilized by the host (Shabat et al., 2016; Lopes et al., 2021). In fact, metabolism performed by the rumen microbiome provides the host with up to 70% of its energy and 90% of its daily protein requirements for body maintenance, growth, production, and reproduction (Bergman, 1990; Flint and Bayer, 2008). However, although the rumen microbiome performs these important functions, it also has drawbacks since ruminal fermentation of feed produces dihydrogen (H_2_) as a metabolic product, which is used by methanogenic archaea to reduce CO_2_ to methane (Kelly et al., 2022). The decrease in H_2_ helps maintain the rumen pH within safe levels and acts as the last electron acceptor in the fermentation process. However, this poses an economic and environmental concern because it represents a significant loss (up to 12%) in the energy from the ruminants’ diet, and it leads to the formation of methane, a greenhouse gas, that contributes to climate change (Johnson and Johnson, 1995; Kelly et al., 2022).

Brazil is the second largest producer of beef in the world, with pure and crossbred Nelore accounting for more than 90% of the Brazilian beef cattle herds [Associação Brasileira de Criadores de Zebuínos (ABCZ), 2023]. Since cattle nutrition can account for up to 85% of the production costs, there is an interest in finding alternatives to make this production system more profitable and sustainable (Thornton, 2010). The usage of by-products in the food production system is an alternative to reduce costs, while it also increases the food system’s circularity by enhancing the use of resources and reducing food–feed competition (Sandström et al., 2022). Recently, 16S rRNA gene sequence data was published by our group showing differences in the microbiome profile of Brazilian Nelore bulls fed a conventional diet or a diet based on agricultural by-products (Andrade et al., 2022). Although the article found taxonomic differences between the dietary groups and associated bacterial amplicon sequence variants (ASVs) to methane emission and feed efficiency traits, substantial knowledge gaps remain, particularly regarding the functional potential of the microbes that compose these microbiomes. Thus, a comprehensive characterization of the microbial metabolism in the Nelore GIT and its association with the host’s phenotypes is essential to unveil how modulation of the bovine microbiome can improve production traits. However, to date, few studies have described the microbiome of Brazilian Nelore cattle, and even fewer considered its relationship with livestock-relevant phenotypes (de Oliveira et al., 2013; Lopes et al., 2019; Andrade et al., 2020, 2022; Lopes et al., 2021).

Therefore, to address these knowledge gaps, here, we performed a more in-depth study by applying shotgun metagenomics, a more robust approach that is free of biases associated with primer selection or PCR amplification irregularities. We explored the rumen and fecal microbiome of the Brazilian Nelore bulls previously studied through 16S rRNA amplicons (Andrade et al., 2022), but now adding new knowledge layers on the relationship of the Nelore microbiome composition and functionality with diet and production traits. The knowledge generated here has the potential to contribute to the development and implementation of feeding and biotechnological strategies to reduce the environmental impact of livestock and improve the efficiency of animal production.

## 2 Materials and methods

### 2.1 Animals and sample collection

The experimental population consisted of 52 Nelore steers (*Bos indicus*) born in 2014. This cohort was divided into two groups based on their diet. The first group (Conventional group, n = 26) received a diet containing corn silage (72.8%), soybean meal (3.06%), corn grains (21.4%), protected fat (1.19%), urea (0.59%) and a mineral mixture (Confinatto N235 Agroceres Multimix®; 0.91%). The second group (By-product group, *n* = 26) received a diet containing corn silage (57.3%), peanut meal (4.7%), fat corn germ (22.59%), citrus pulp (13.96%), urea (0.30%) and the same mineral mixture (Confinatto N235 Agroceres Multimix®; 1.15%). The animals received mineral supplements, active dry yeast, virginiamycin, and monensin in both treatments. The composition and nutritional levels of both dietary treatments are available in Supplementary Table S1.

The experiment was conducted at the feedlot facility of “Embrapa Pecuária Sudeste.” Due to initial body weight heterogeneity, heavier and lighter animals were evenly allocated within each diet group. The feedlot experiment lasted for 90 or 121 days, depending on the initial body weight, which included 10 days for animal adaptation to the feedlot, 29 days for heavy animals and 55 days for light animals in the growing phase, and 51 days for heavy animals and 56 days for light animals in the finishing phase. The animals were sent to slaughter at 23–24 months of age after fasting from food and water for 16 h, according to the current Brazilian Ministry of Agriculture, Livestock and Food Supply (MAPA) regulations. Approximately 10 g of stool was obtained from each animal 2 weeks before slaughter, and 50 mL of rumen content was collected immediately after slaughter. Rumen samples were immediately frozen in liquid nitrogen and stored at −80°C before DNA extraction, while stool samples were kept in ice for approximately 2 h before being stored at −80°C.

All experimental procedures followed the Animal Welfare and Humane Slaughter guidelines and were approved by EMBRAPA Livestock Science Ethics Committee on Animal Experimentation, São Carlos, São Paulo, Brazil (Protocol No. 09/2016).

### 2.2 Phenotype data

Automatic feeding systems (GrowSafe Systems Ltd., Airdrie, Alberta, Canada) were used to collect data regarding daily food consumption. Enteric methane emissions were measured during the finishing period in the feedlot using the GreenFeed automated system (C-lock Inc., Rapid City, SD, United States). Animals were weighed at the beginning and end of the feedlot period after a fasting (16 h) period and at 28-day intervals during this period without fasting for live weight gain monitoring. All animal data used in this study are available in Supplementary Table S2.

As described previously (Andrade et al., 2022), Dry Matter Intake (DMI, kg/d) of each animal was obtained automatically with the Growsafe platform, and Average Daily Gain (ADG, kg/d) was estimated by linear regression of Body Weight (BW) on days in the feedlot. Residual Feed Intake (RFI, kg/d) was computed as the residuals from the regression of DMI on mid-test body weight BW^0.75^ and ADG (Koch et al., 1963). The Residual Methane Emission (RME) of each animal was obtained by the regression of methane emission on individual DMI (Herd et al., 2014; Donoghue et al., 2016).

Two correction models were implemented to the RFI and RME indexes described above. One model considered the 52 animals and the contemporary group (CG), which was defined as the weighing groups (heavy-weight and light-weight animals) and slaughter groups (groups of animals slaughtered on different days). The second model considered the 26 animals on each diet separately and corrected for the CG. These corrections were implemented by the MIXED procedure of the SAS statistical program (SAS Institute, Cary, NC, United States, 2011), including the CG groups as fixed effects.

The calculation of RME used by both models was performed using the following equation:


$$MEi=\beta_{0}+\beta_{1}\;\left(DMI_{i}\right)+RME_{i}$$

Where ME_i_ is the methane emission observed for animal i; DMI_i_ is the dry matter intake predicted for animal i; β_0_ is the regression intercept; β_1_ is the partial regression coefficient of DMI; and RME_i_ of animal i is the residual methane emission as proposed by Herd et al. (2014) and Donoghue et al. (2016), where it is referred to as RMP_R_. For the first model, β_0_ = 193,94 and β_1_ = −2,0332, while for the second model, β_0_ = 215,72 and β_1_ = −3,2,782 considering the conventional diet and β_0_ = 162,17 and β_1_ = − 0,3,083 considering the by-products diet.

### 2.3 Nucleic acid extraction and metagenomic shotgun sequencing

Total DNA was extracted from 150 mg of homogenized rumen content samples and from the same mass of fecal samples using the Quick-DNA™ Fecal/Soil Microbe Miniprep Kit (ZYMO Research Corp., Irvine, CA), according to the manufacturer’s instructions and stored at −80°C until sequencing. Metagenomic libraries were constructed with Illumina DNA Prep Kit following the standard protocols. The purified libraries were sequenced on an Illumina NextSeq sequencer platform (ESALQ Genomics Center, Piracicaba, SP, Brazil) using the NextSeq P3 flowcell 300 cycles (Illumina). A total of 5,361,477,858 paired-end 2 × 150 bp reads were obtained from 104 samples of 52 animals, with an average of ~51.5 million reads per metagenome (Supplementary Table S2).

### 2.4 Bioinformatics processing and statistical analysis

The generated reads were trimmed and filtered using Trimmomatic (Bolger et al., 2014) with the options “ILLUMINACLIP:adapters.fasta:2:30:10 LEADING:3 TRAILING:3 SLIDINGWINDOW:4:15 MINLEN:30.” The remaining reads were mapped to the *Bos indicus x Bos taurus (hybrid cattle)* host reference genome UOA_Brahman_1 (RefSeq assembly accession: GCF_003369695.1) using Bowtie2 (Langmead and Salzberg, 2012) with default parameters. The resultant filtered reads (5,221,576,952 reads) were used in further analysis (Supplementary Table S2).

Taxonomic classification of each metagenome was performed using Kraken2 (Wood et al., 2019) with the options “--paired --gzip-compressed --threads 24 --use-names --use-mpa-style,” and using all genomes of bacteria and archaea in RefSeq from NCBI (O’Leary et al., 2016) as reference. Functional classification was performed using SUPER-FOCUS (Silva et al., 2016) with default parameters, and using the gene families that compose the SEED database (Overbeek et al., 2005) as reference. In this database, the functional roles of each gene family are categorized into subsystems with four levels of resolution. Level 1 represents the broader categories, which are divided into subcategories (level 2), level 3 represents the pathway(s) in which a feature is involved and level 4 is the actual function assigned to a feature.

For general data manipulation and statistical analysis, we employed the vegan and phyloseq packages in R version 4.2.1. Shannon index of alpha-diversity was estimated for each metagenome, with the pairwise Wilcoxon test being used for statistical difference evaluation between sites and treatment groups. Beta-diversity was determined with Principal coordinate analysis (PCoA) generated with Bray–Curtis dissimilarity distances. Permutational multivariate analysis of variance (PERMANOVA) was performed with 999 permutations to estimate a *p*-value for differences among the groups analyzed.

To avoid potential microbiome variations related to the contemporary groups (defined as the weighing groups and slaughter groups), taxonomic abundances and gene functions (level 3) abundances tables were corrected using MMUPHin (Meta-Analysis Methods with a Uniform Pipeline for Heterogeneity in microbiome studies; Ma et al., 2022) in the batch correction model (adjust_batch) with default parameters. Next, a linear discriminant analysis (LDA) was performed using LEfSe (Segata et al., 2011) to detect features that characterize the differences between the dietary groups. For LEfSe analysis, the Kruskal–Wallis test (alpha value ≤ 0.05) and LDA score of >2.0 were used as thresholds.

Linear regression analysis between the Shannon diversity indexes and the animal phenotypes (RFI and RME) was also performed, with *p*-value < 0.05 used to consider the relationship between them as significant. This analysis was performed separately for each group of samples (rumen or feces). The first analysis considered the Shannon diversity indexes of the 52 animals and phenotype indices generated with the model considering the CG as fixed effect and the dietary treatments as a β3 variable.


$$\mathrm{lm}1:\mathrm{Phenotype}\left(RFI:GC\;\mathrm{or}\;RME:GC\right)\sim \mathrm{Shannon}\;\mathrm{index}$$

To evaluate these associations taking into account the effect of diet, the variable Diet and its interaction with the Shannon index was added to the formula, whereas the phenotypes were not corrected for these factors:


$$\begin{array}{l} \mathrm{lm}2:\mathrm{Phenotype}\left(RFI:GC\;\mathrm{or}\;RME:GC\right)\sim \mathrm{Shannon}\;\mathrm{index}\\ +\mathrm{Diet}+\mathrm{Shannon}\;\mathrm{index}:\mathrm{Diet} \end{array}$$

To evaluate whether the individual diets had different effects on these associations, separate analyses considering the 26 animals on each diet and phenotype indexes only corrected for CG were performed using the following formula:


$$\begin{array}{l} \mathrm{lm}3:\mathrm{Phenotype}\left(RFI:by:\mathrm{diet}\;\mathrm{or}\;RME:by:\mathrm{diet}\right)\\ \sim \mathrm{Shannon}\;\mathrm{index} \end{array}$$

Associations between microbial abundances and the animal phenotypes (RFI and RME) were performed using MaAsLin 2 (Microbiome Multivariable Associations with Linear Models; Mallick et al., 2021) with the options “min_abundance = 0.05, min_prevalence = 0.5, max_significance = 0.05, normalization = “CLR,” transform = “None,” analysis_method = “LM,” correction = “BH,” standardize = FALSE.” These analyses were performed separately for rumen and feces without separating dietary groups and therefore, we used the phenotype indices RFI_GC and RME_GC. The features were pre-filtered for ≥50% prevalence, normalized using Centered Log-Ratio (CLR) transformation with Diet and each phenotype being applied as fixed effects.

To survey the functional potential of the taxa significantly associated with RFI and RME through MaAsLin 2, we retrieved the identifiers of the reads that had previously been assigned to these taxa (by Kraken) and extracted the potential function classification that had been assigned to them (by SUPER-FOCUS). In order to investigate the functional similarities among these taxa, we built a matrix to determine the Bray–Curtis dissimilarity between the taxa and generated a PCoA.

## 3 Results

### 3.1 Diversity and composition of the Nelore microbiomes

**Figure 1:**
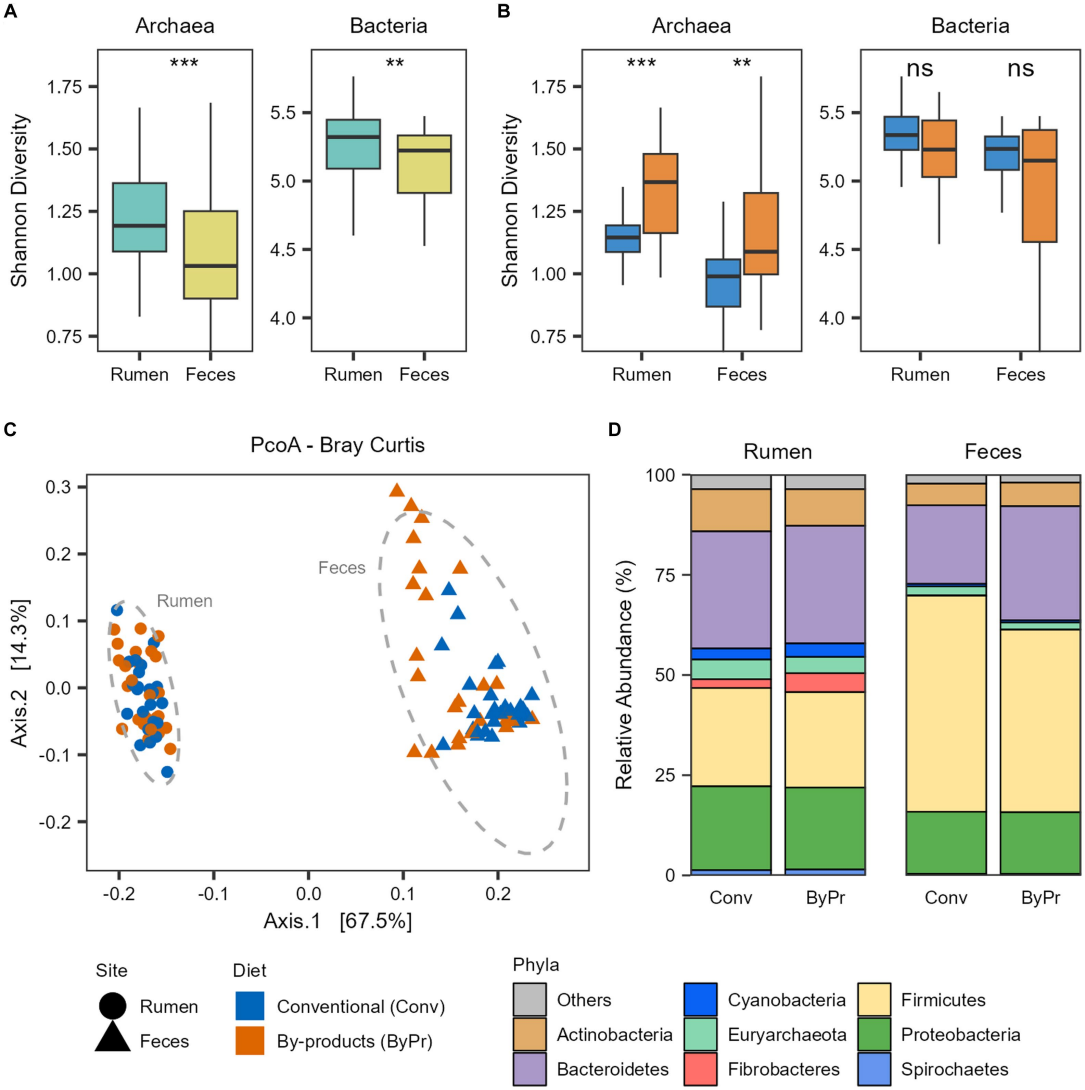
Taxonomic diversity and composition of the Nelore microbiomes. Alpha diversity of the Nelore microbiomes was calculated with Shannon index on genus-level taxa tables. ns, not significant; **p < 0.01, ***p < 0.001 (FDR-corrected Wilcoxon test). Bacterial and archaeal diversity considering the **(A)** GIT samples and **(B)** the two treatment groups. **(C)** Principal coordinate analysis (PCoA) generated with Bray–Curtis dissimilarity distances of microbial genera identified in the metagenomes. **(D)** Relative abundance of the main phyla (whose mean abundance is greater than 1%) in the ruminal and fecal microbiomes of the two treatment groups.

To unravel the ruminal and fecal microbiome diversity of the animals studied, we performed alpha- and beta-diversity analyses based on the bacterial and archaeal genus level identified with Kraken (Supplementary Table S3). Alpha diversity was measured using Shannon’s index and demonstrated that the microbial diversity identified in the Nelore microbiomes varied between the GIT samples. The rumen microbiome showed significantly higher bacterial and archaeal diversity than the fecal microbiome (FDR-corrected Wilcoxon test: *p* < 0.05; Figure 1A). The bacterial and archaeal diversities in the microbiomes of the two treatment groups were also compared (Figure 1B). The ruminal and fecal microbiomes of the animals fed the by-products diet had a significantly higher archaea diversity than those fed the conventional diet (FDR-corrected Wilcoxon test: *p* < 0.05; Figure 1B). Regarding the bacterial diversity, no significant differences were observed between the microbiomes of the treatment groups (FDR-corrected Wilcoxon test: *p* > 0.05; Figure 1B). Bray-Curtis analysis showed clear segregation between the Nelore ruminal and fecal microbiome composition (PERMANOVA, pvalFDR = 0.001; Figure 1C), with the fecal microbiome being more dispersed than the ruminal microbiome (PERMDISP2 Pr = 0.001).

The taxonomic profile of the Brazilian Nelore gut microbiome from the rumen and fecal samples is mainly composed of Bacteria (mean ± sd: 96.6 ± 1.50%), with a small fraction of reads corresponding to Archaea (mean ± sd: 3.22 ± 1.46%). The microbiomes were compared at both phylum and genus levels to identify which bacterial and archaeal taxa differentiate between the GIT samples and dietary groups. The most abundant phylum of both ruminal and fecal microbiomes were *Bacteroidetes*, *Firmicutes*, *Proteobacteria*, and *Actinobacteria*. Although both samples were mostly characterized by these phyla, apparent differences in their abundance were observed between the rumen and fecal microbiomes (Figure 1D). Moreover, *Fibrobacteres*, *Cyanobacteria*, and *Spirochaetes* also showed a higher abundance in rumen microbiomes. In contrast, these three phyla seem to be primarily depleted in fecal microbiomes. Regarding Archaea, the phylum *Euryarchaeota* is the most abundant in both ruminal and fecal microbiomes (Figure 1D); however, it is significantly more abundant in the rumen (FDR-corrected Wilcoxon test: *p* < 0.05).

Comparing the dietary groups, *Actinobacteria* and *Euryarchaeota* were significantly more abundant in the Conventional group, while the phylum *Fibrobacteres* was significantly more abundant in the By-products group (FDR-corrected Wilcoxon test: p < 0.05) in the rumen microbiome. In the fecal microbiome, *Firmicutes* and *Euryarchaeota* were significantly more abundant in the Conventional group, while *Bacteroidetes* was significantly more abundant in the By-products group (FDR-corrected Wilcoxon test: *p* < 0.05; Figure 1D).

**Figure 2:**
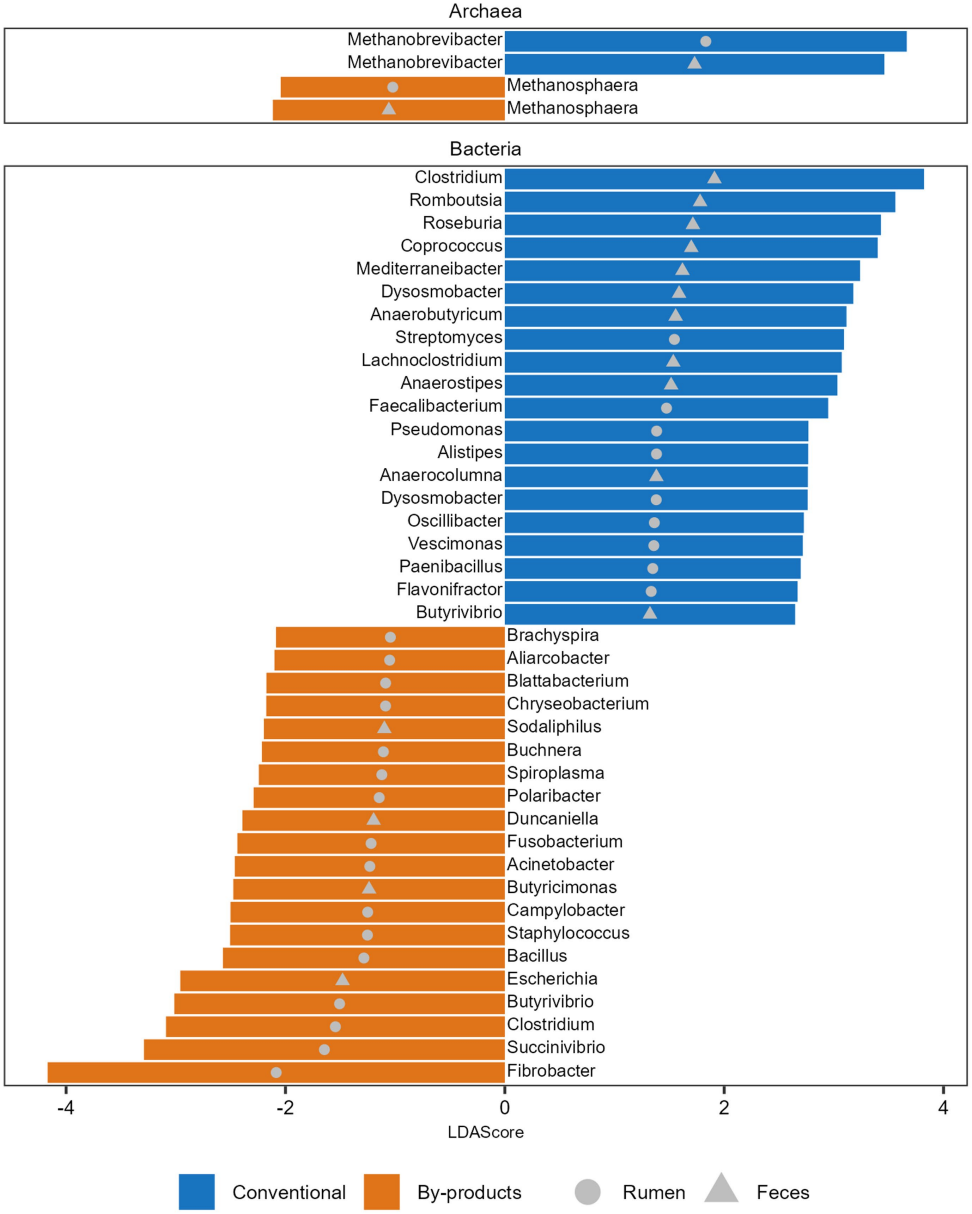
Microbial taxa significantly more abundant in each diet. Histogram of the Linear discriminant analysis (LDA) scores illustrating the differentially abundant archaeal genera and top 20 bacterial genera in the ruminal and fecal microbiomes of the Nelore animals fed two diets: Conventional (positive LDA score) or by-products (negative LDA score) diet. Only LDA scores (log 10) > 2 were considered.

We also observed that the microbial genera enriched in the animals fed different diets varied in both GIT samples studied. We identified 89 differentially abundant genera between the dietary groups in the rumen and feces (Supplementary Table S4). The 20 genera with higher LDA are presented in Figure 2. The archaeal genus *Methanobrevibacter*, which comprises 73%–92% of the archaeal reads in all metagenomes, was enriched in the rumen and feces of the animals fed the conventional diet (Figure 2). In parallel, *Methanosphaera*, the second most abundant archaeal genus, comprising 1.2%–8% of the archaeal reads in all metagenomes, was enriched in the rumen and feces of animals fed the by-products diet (Figure 2). Regarding bacteria, the most prominent genera identified in the rumen of the Conventional group were *Streptomyces*, *Faecalibacterium,* and *Pseudomonas,* and in the feces were *Clostridium*, *Romboutsia,* and *Roseburia*. In the By-products group, the most prominent bacterial genera identified in the rumen of animals from the By-products group were *Fibrobacter*, *Succinivibrio,* and *Clostridium,* and in the feces were *Escherichia*, *Butyricimonas,* and *Duncaniella* (Figure 2).

### 3.2 Nelore microbiome functional potential

We classified the metagenomic reads to gene families to explore the ruminal and fecal microbiome’s functional potential based on the SEED database^1^ information (Supplementary Table S5). Firstly, beta-diversity analysis based on the abundance of functions at the subsystem level 3 (which represents their pathways) showed a similar pattern to the one observed with the genera abundance, with clear segregation between ruminal and fecal metagenomes (PERMANOVA, pvalFDR = 0.001; Supplementary Figure S1).

**Figure 3:**
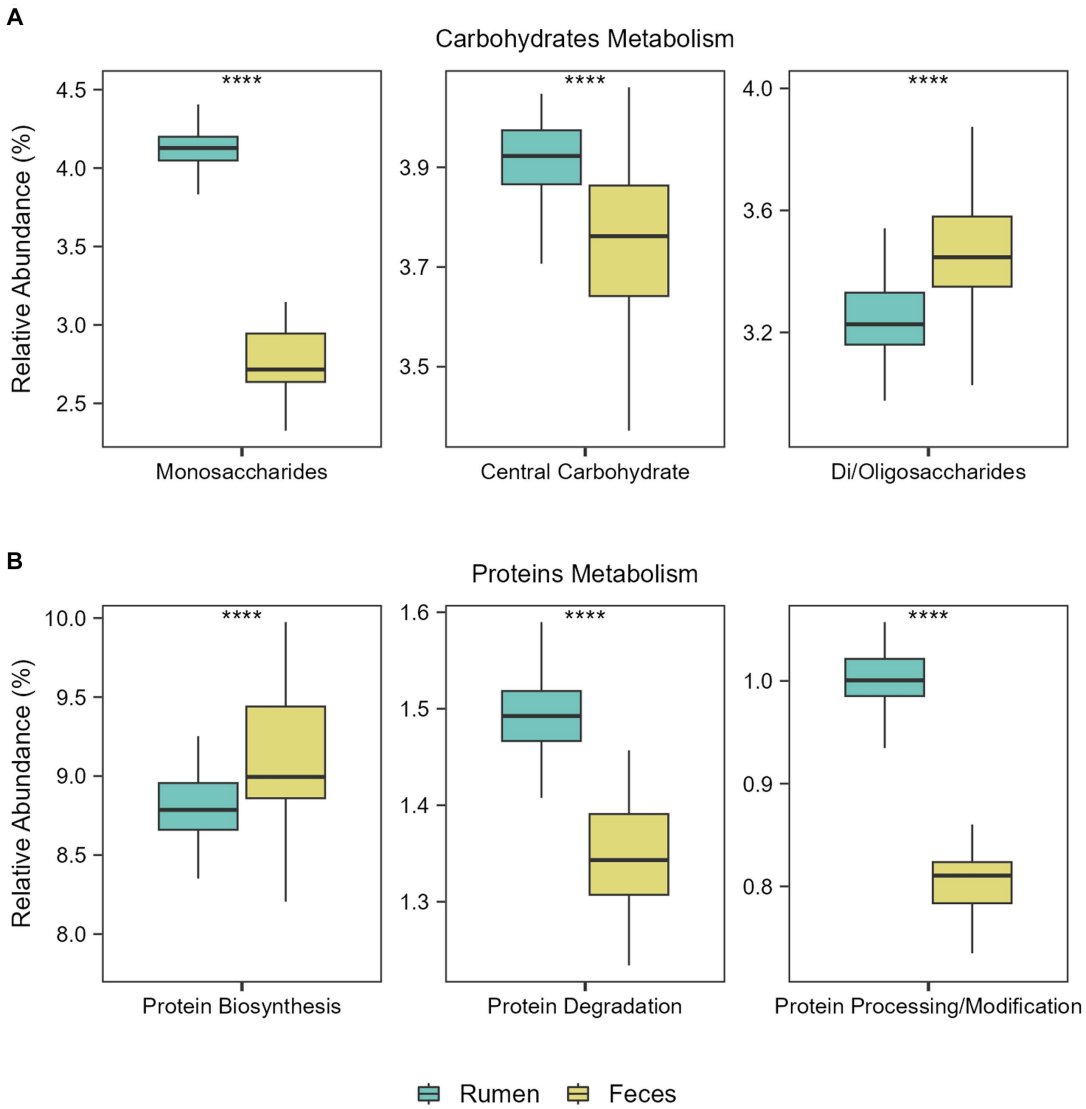
Functional metabolic differences between ruminal and fecal microbiomes. Boxplots showing the abundance of the main functions related to **(A)** Carbohydrates Metabolism and **(B)** Proteins Metabolism in the rumen and feces of the Nelore animals studied. Analyses were performed considering the Level 1 SEED category. ^****^p ≤ 0.0001 (FDR-corrected Wilcoxon test).

Then, we explored the main functions of rumen and fecal microbiomes regardless of the diets. Considering subsystem level 1 (broader category), the most abundant functions in both environments were related to the metabolism of carbohydrates and proteins. Considering subsystem level 2, significant differences in the abundance of the main functions related to carbohydrate and protein metabolism in the ruminal and fecal microbiomes were observed: Monosaccharide and central carbohydrate metabolism were the main functions in the rumen microbiome, while central carbohydrate metabolism and di−/oligosaccharides metabolism were the main functions in the fecal microbiome (FDR-corrected Wilcoxon test: *p* ≤ 0.0001; Figure 3A). Regarding functions related to protein metabolism, protein biosynthesis was the main function in the fecal microbiome, while protein degradation and protein processing/modification were the main functions in the rumen microbiome (FDR-corrected Wilcoxon test: *p* ≤ 0.0001; Figure 3B).

**Figure 4:**
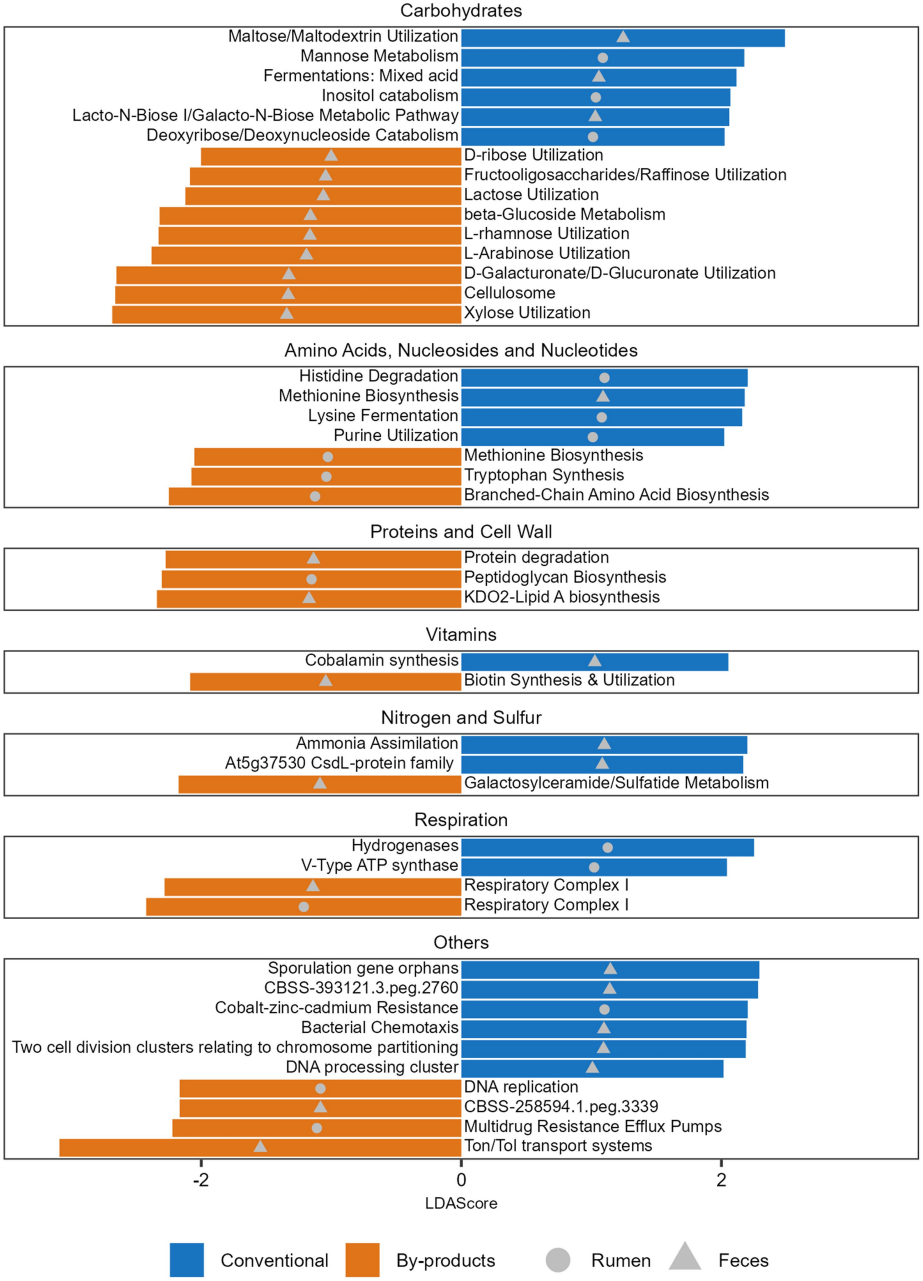
Functions significantly more abundant in each diet. Histogram of the LDA Scores illustrating the differential abundant functions in the ruminal and fecal microbiomes of the Nelore animals fed two diets: Conventional (positive LDA score) or by-products (negative LDA score) diet. Analyses were performed considering the Level 3 SEED category. Only LDA scores (log 10) > 2 were considered.

When comparing the functional profiles of both GIT samples considering the diets, we observed that the level 3 functions enriched in the animals fed different diets varied (Supplementary Table S6) and the main differences were related to the metabolism of carbohydrates. Within the functions related to monosaccharides metabolism, it was possible to observe that mannose metabolism, as well as deoxyribose and deoxynucleoside catabolism, were significantly enriched in the Conventional group, while functions such as xylose, d-galacturonate, and d-glucuronate utilization were significantly enriched in the By-products group (Figure 4). Within the functions related to di/oligosaccharides and polysaccharides, maltose and maltodextrin utilization were enriched in the Conventional group, while fructooligosaccharides, raffinose and lactose utilization and cellulosome were enriched in the By-products group. Among the functions related to the metabolism of sugar alcohols, we observed a significant enrichment of Inositol catabolism in the rumen microbiome of the Conventional group. Differences related to the biosynthesis or degradation of amino acids, nucleosides and nucleotides were also observed, specifically histidine, lysine, methionine and purine in the Conventional group; and tryptophan, branched-chain amino acid and methionine in the By-products group (Figure 4). Other differences are related to the biosynthesis and degradation of proteins and components of cell walls, metabolism of vitamins, nitrogen and sulfur, but also respiration and others.

### 3.3 Associations between microbiome taxa diversity and phenotypes

We investigated the relationship between microbiomes and phenotypes, focusing on indices for feed efficiency (RFI) and methane emission (RME). The RFI values for the studied animals in the By-products group ranged from −3.1530 to 4.3410, with a mean of 0.2025 and a standard deviation of 1.8624, while in the Conventional group, they ranged from −2.2310 to 2.2210, with a mean of −0.2025 and a standard deviation of 1.1031. The RME values in the By-products group ranged from −62.475 to 43.762, with a mean of −7.950 and a standard deviation of 21.9717, and in the Conventional group, they ranged from −33.072 to 59.324, with a mean of 7.950 and a standard deviation of 22.1364. In these indices, lower RFI values denote greater feed efficiency, whereas higher RFI values denote lower feed efficiency. Inversely, lower RME values indicate reduced methane emissions, while higher RME values indicate greater emissions. There was no statistically significant differences in the comparison of the phenotypic values between the treatment groups (t-test: *p*-value > 0.05).

**Figure 5:**
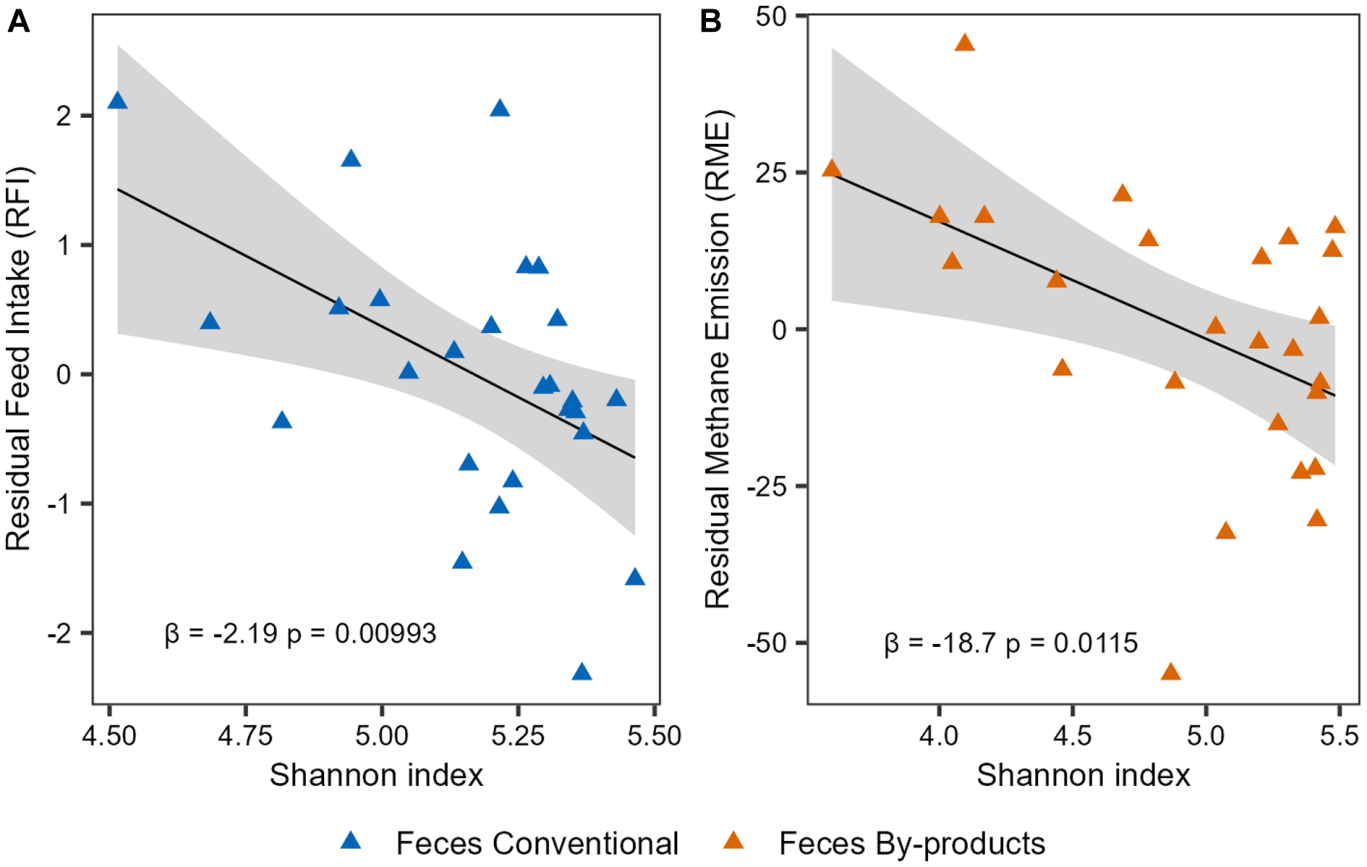
Microbial diversity association with feed efficiency and methane emission indexes. Linear regression analysis expressing the relationship between Shannon diversity with **(A)** residual feed intake (RFI) in the feces of animals fed the conventional diet and with **(B)** residual methane emission (RME) in the feces of the animals fed the by-products diet. The colors of the points represent the different diets: the conventional diet in blue and the by-products diet in orange. The slope β1 and the association’s significant *p*-value (*p* < 0.05) are shown.

To investigate the association of the phenotypes and microbiome alpha diversity, we performed linear regression analyses for the Shannon index and the phenotypes indices (RFI and RME). First, we performed the analysis considering the 52 animals and using the phenotype indices corrected for CG. No significant association was observed between microbial diversity and RFI or RME in both rumen and feces (*p*-value > 0.05). To further investigate these results, we performed a multiple linear regression analysis now taking into account the effect of diet. For the RFI phenotype, a significant association was observed in the interaction between Shannon index and diet (β = 2.903, *p*-value = 0.0395) in feces, suggesting that the effect of Shannon index on feed efficiency of cattle depends on their diet. As for the RME phenotype, when the effects of diet were taken into account, an association between the By-products diet and the phenotypic index was observed (β = 83.237, *p*-value = 0.0317) in feces. Therefore, to understand whether the individual diets had different effects on these associations, we performed separate analyses considering the 26 animals on each diet and phenotype indexes only corrected for CG. Our analysis revealed a negative correlation between the Shannon diversity index and RFI in the feces of the Conventional group (β = −2.187, *p*-value = 0.00993; Figure 5A) and a negative correlation between the Shannon diversity index and RME in the feces of the By-products group (β = −18.715, *p*-value = 0.0115; Figure 5B). Therefore, microbial (bacterial and archaeal) diversity was higher in the feces of feed-efficient animals (low-RFI) fed conventional diet, whereas it was higher in animals that emit less methane (low-RME) and were fed by-products diet.

### 3.4 Taxa associated with RFI and their potential functions

**Figure 6:**
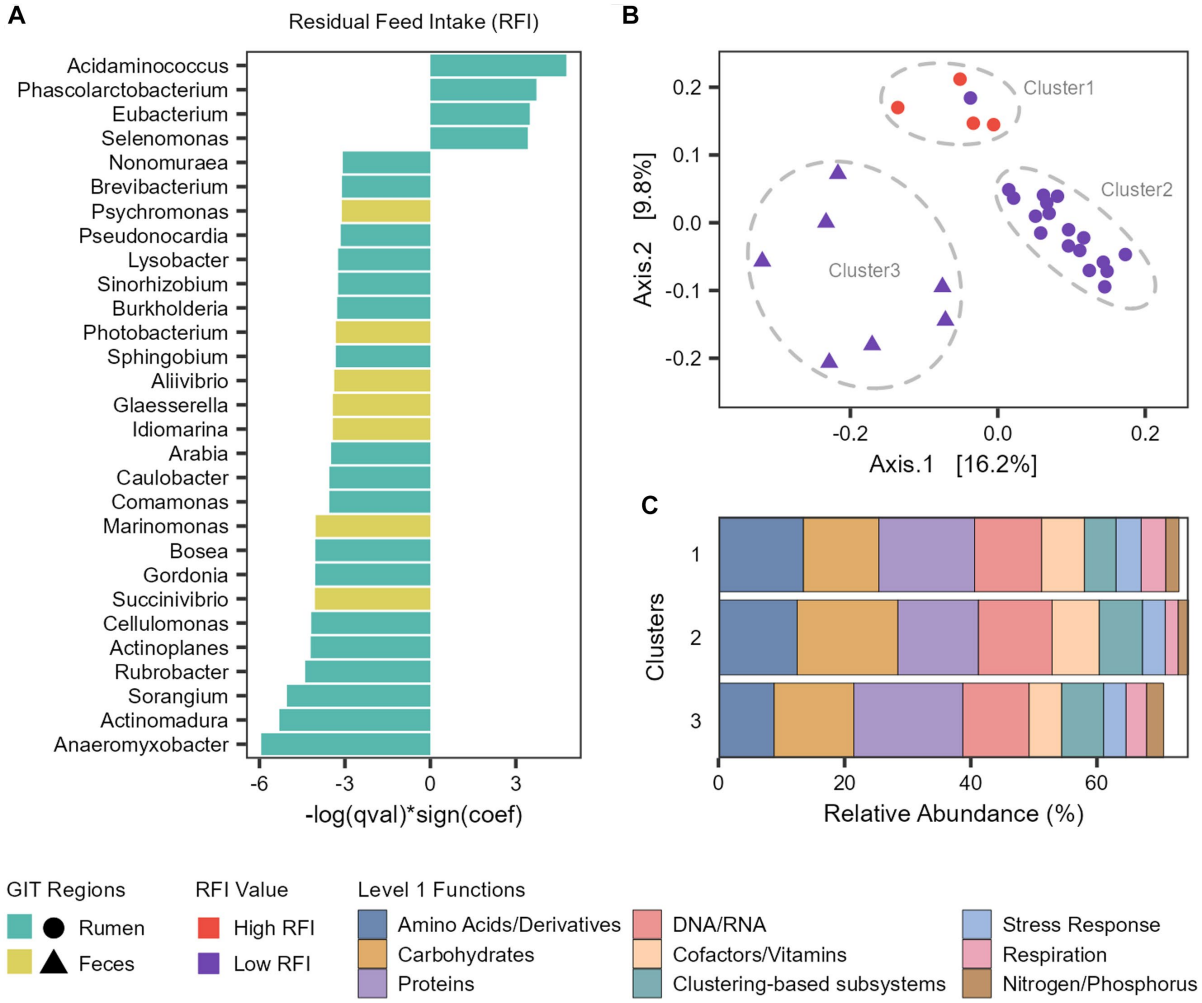
Taxonomic and functional features associated with feed efficiency. **(A)** Microbial genera significantly associated with Residual Feed Intake (RFI). **(B)** Principal coordinate analysis (PCoA) generated with Bray–Curtis dissimilarity based on the functional profile (Level 3 SEED category) of each taxon significantly associated with RFI. **(C)** Barplot representing the mean relative abundance (percentage) of the main level 1 functions from each cluster.

**Figure 7:**
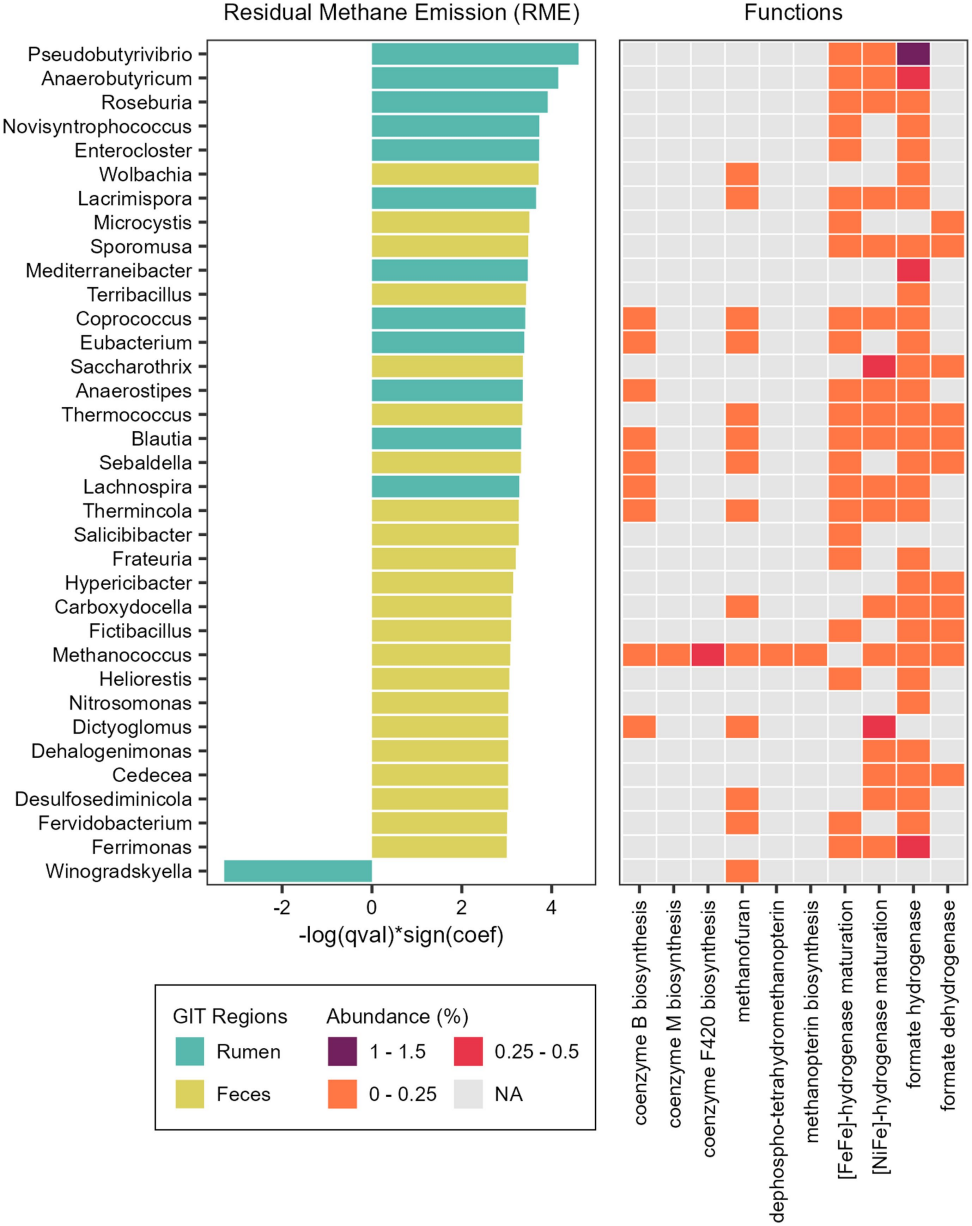
Taxonomic and functional features associated with methane emission. Microbial genera significantly associated with Residual Methane Emission (RME) and heatmap showing the relative abundance of specific functions (Level 3 SEED category) attributed to the reads classified as each taxon significantly associated with RME.

To associate microbial abundances to the phenotypes, we performed a multivariable linear mixed model with MaAsLin 2 (Supplementary Table S7). In the rumen microbiome, the abundance of *Acidaminococcus*, *Phascolarctobacterium, Eubacterium* and *Selenomonas* genera were significantly associated with an increase of the RFI values (inefficient cattle) and 14 other genera were significantly associated with a decrease of the RFI values (efficient cattle), with the more prominent being *Anaeromyxobacter*, *Actinomadura*, *Sorangium*, and *Rubrobacter* (Figure 6A). In the fecal microbiome, we only identified genera significantly associated with a decrease of the RFI values (efficient cattle), with *Succinivibrio* and *Marinomonas* being the more prominent (Figure 7A).

To explore the functional potential of the taxa significantly associated with RFI, we retrieved the reads assigned to each taxon and analyzed which functions (level 3) were attributed to them in the functional analysis (Supplementary Table S8). Next, we compared the functional profile among the taxa with a PCoA generated with Bray–Curtis dissimilarity (Figure 6B). With this, we observed that the taxa were segregated into three clusters. Cluster 1 is composed of the four ruminal taxa associated with an increase of RFI (Less efficient cattle) and *Arabia* (which was associated with more efficient cattle), Cluster 2 comprises all the other ruminal taxa associated with a decrease of RFI (More efficient cattle), while Cluster 3 is composed of fecal taxa associated with a decrease of RFI (Figure 6B).

Metabolism of amino acids, carbohydrates, proteins and DNA/RNA are the main subsystems related with the three clusters (Figure 6C). However, there are differences in the abundances and functions that compose them. We focused our analysis on the differences between clusters 1 (High RFI and *Arabia*) and 2 (Low RFI) since they both comprise ruminal taxa. Regarding carbohydrates metabolism, Cluster 2 exhibits significantly higher levels of functions related to monosaccharides, di−/oligosaccharides and amino sugars metabolism at level 2 and cellulosome, xylose utilization and acetyl-coA fermentation to butyrate at level 3 (FDR-corrected Wilcoxon test: *p* < 0.05; Supplementary Figure S2). Regarding proteins metabolism, the main functions in Cluster 1 were related to the biosynthesis of proteins, significantly more abundant than in Cluster 2, while the main functions in Cluster 2 were related to the degradation of proteins, significantly more abundant than in Cluster 1 (FDR-corrected Wilcoxon test: *p* < 0.05; Supplementary Figure S2). Cluster 2 also shows significant enrichment in functions related to fatty acid catabolism, while Cluster 1 exhibits enrichment in ATP synthases (FDR-corrected Wilcoxon test: *p* < 0.05; Supplementary Figure S2).

### 3.5 Taxa associated with RME and their potential functions

We also found associations between microbial abundances and methane emission (Supplementary Table S7). In the rumen microbiome, only the genus *Winogradskyella* was significantly associated with a decrease of RME values (lower methane emission), while 12 other genera were significantly associated with an increase of RME (higher methane emission), with the more prominent being *Pseudobutyrivibrio*, *Anaerobutyricum*, and *Roseburia* (Figure 7). In the fecal microbiome, 22 genera were associated with an increase of RME (higher methane emission), with *Wolbachia*, *Microcystis*, *Terribacillus* and *Sporomusa* being the more prominent, and none were associated with a decrease of RME (lower methane emission). Besides these bacterial taxa, the methanogenic archaea *Methanococcus* and the thermophilic archaea *Thermococcus* were also significantly associated with an increase of the RME (higher methane emission) in the fecal microbiome (Figure 7).

To explore the functional potential of the taxa significantly associated with RME, we performed the same steps done for the RFI phenotype (Supplementary Table S9). With the PCoA, we could observe a higher dispersion of the taxa from the fecal microbiome, indicating a quite diverse functional profile. The taxa from the ruminal microbiome that are positively associated with RME grouped together, while the single taxon negatively associated with RME could be found near the fecal taxa associated with an increase of RME (Supplementary Figure S3).

Considering the broader functional category (level 1) it was possible to identify differences between taxa associated with RME. The main functions of the ruminal genus *Winogradskyella*, significantly associated with lower methane emission, were related to Protein Metabolism, DNA Metabolism and Cell Wall and Capsule. The main functions of the ruminal taxa significantly associated with a higher methane emission were related to Carbohydrates, Protein Metabolism and Amino Acids and Derivatives. Considering the fecal taxa, the main functions were more diverse and mainly related to metabolism, such as the metabolism of proteins, DNA, RNA and carbohydrates, but also clustering-based subsystems and respiration.

We also focused on functions related to methane production (methanogenesis) and functions that produce substrates with the potential to influence it. We identified hydrogenases and dehydrogenases ([NiFe]-hydrogenase, [FeFe]-hydrogenase, formate hydrogenase and formate dehydrogenase), coenzymes and cofactors (coenzyme M, coenzyme B, methanofuran, methanopterin, F420) in the taxa significantly associated with RME trait (Figure 7).

## 4 Discussion

In this study, we explored the ruminal and fecal microbiome of 52 Brazilian Nelore bulls under two different dietary treatments (Graphical abstract). While most studies focus on ruminal microbiomes, we also included fecal samples since they are more easily accessible than ruminal samples during animal management. In addition, our study also sheds light on the association between the functional potential of these microbial communities and the diet composition of the host. Furthermore, we found associations with feed efficiency and methane emission in the Nelore microbiome. We identified functions that may influence these phenotypes by observing the genetic functional potential of taxa significantly associated with them.

### 4.1 Co-occurrence and variations between the ruminal and fecal microbiomes

Different taxonomic diversity and composition between the rumen and the feces of bulls have been shown in previous studies (de Oliveira et al., 2013; Lopes et al., 2019; Andrade et al., 2020, 2022; Welch et al., 2020; Monteiro et al., 2022). Here, in addition to taxonomic analysis, we emphasize the functional potential of the microbes present in these two environments, showing new insights into how this difference occurs, also considering the effect of different diets, and presenting the main related metabolic pathways. Although functions related to feed degradation were shared across both GIT samples, the abundance of some specific functions differed. Monosaccharide metabolism and protein degradation were significantly more abundant in the rumen microbiome, while di-/oligosaccharides metabolism and protein biosynthesis were significantly more abundant in the fecal microbiome. This goes along with the concept that the rumen microbiome is a major contributor to ruminant digestion, where most of the fermentation of dietary components takes place and is a site of absorption for macromolecules (Monteiro et al., 2022). The lower gut microbiome ferments nutrients that escape degradation and absorption in earlier compartments of the GIT and thus contributes to ~10% of the total metabolizable energy ingested by the animal (Monteiro et al., 2022). Moreover, taxonomical and functional variations in these environments may also be explained by the different physiological conditions (such as the pH and passage rate) and nutrient availability (Henderson et al., 2015).

### 4.2 Taxonomic and functional microbiome profiles associated with diet composition

The GIT microbes perform cellulolytic, amylolytic, and proteolytic functions to digest different feed components. They provide the host with nutrients and energy sources (volatile fatty acids) as well as building blocks (carbohydrates, peptides, lipids; Flint et al., 2012). Since these functions are related to diet, changes in the composition and functions of the microbiomes of animals fed different diets are to be expected. Indeed, this was observed in this study, in which the diets used presented considerable differences in their nutrient composition. The concentrates in the conventional diet (corn grain and soybean meal) were replaced by corn germ oil meal, citrus pulp, and peanut meal in the by-products diet. Therefore, the conventional diet contains twice as much starch, while the by-products diet contains more fiber and fat.

The citrus pulp and corn germ, which are part of the by-products diet, are known to contain relatively large amounts of polysaccharides, such as cellulose and hemicellulose (Bampidis and Robinson, 2006; Li et al., 2022). Some microbes produce cellulosomes and/or use hydrolases to break the β-glucosidic linkages that bound the glucose monomers in cellulose or the d-fructose monomers in fructooligosaccharides (oligofructans), both being ubiquitous in fruits and vegetables (Jovanovic-Malinovska et al., 2014; Artzi et al., 2017). Accordingly, functions related to cellulosomes, β-glucoside metabolism, and utilization of fructooligosaccharides were significantly enriched in the feces of the by-products group. In agreement with this, *Clostridium* was an enriched genus in the rumen of the by-product group, and some cellulosome-producing *Clostridium* species (Artzi et al., 2017) were found in the metagenomes, such as *C. acetobutylicum*, *C. bornimense*, *C. cellulovorans*, and *C. saccharoperbutylacetonicum*.

Moreover, the main hemicelluloses in the cell wall of edible fruits are xyloglucans and xylans, which are composed of xylose and l-arabinose (Flint et al., 2012; Artzi et al., 2017). Functions related to the utilization of these two pentoses were also enriched in the by-product group. Additionally, the citrus pulp contains pectins, which are polysaccharides rich in galacturonic acid (d-galacturonate) and also glucuronic acid (d-glucuronate), rhamnose, and arabinose, in significant amounts (Bampidis and Robinson, 2006; Mohnen, 2008). Correspondingly, functions related to the utilization of these four compounds were enriched in the microbiomes of the by-products diet group as well. Besides those, raffinose and d-ribose utilization were significantly enriched in the feces of the by-products group. Raffinose is a trisaccharide composed of galactose, fructose, and glucose and is found in corn and peanuts (Pattee et al., 2000; Li et al., 2017), two components of the by-products diet. D-ribose is one of the most abundant pentose monosaccharides found in nature and is a product of the digestion of nucleic acid from food sources or dead bacterial cells (Pokusaeva et al., 2010).

In the microbiomes of the conventional group, we observed a significant enrichment of functions directly related to the metabolism of specific components in their diet as well. Starch is the major component of corn (Yu and Moon, 2021), which constitutes most of the conventional diet (37.73%). Amylolytic bacteria are able to degrade starch for possessing extracellular amylases that hydrolyze amylose and amylopectin (starch components), producing maltose, maltotriose, glucose, and a mixture of dextrins (Flint et al., 2012). In parallel, soybean meal is low in starch but is high in β-mannan, which is composed of repeating units of mannose, with galactose and/or glucose being often found attached to its backbone (Lin et al., 2011; Sharma et al., 2014). The functions of maltose and maltodextrin utilization and mannose metabolism were significantly more abundant in the conventional group. An example of bacteria that produces high amounts of maltose from starch is *Streptomyces* sp. *IMD 2679* (McMahon et al., 1999), being this genus identified as significantly enriched in the ruminal microbiome of the conventional group.

Corn and soybeans store phosphorus in the form of phytate salts of phytic acid (inositol hexakisphophate), which accounts for 60% to 80% of the total P content in cereal grains (Raboy et al., 2000; Redekar et al., 2020). The catabolism of this acid ensures the supply of phosphorus in the rumen, which is essential for the microbes of this environment to perform adequate fermentation processes and growth (Mickdam et al., 2017). And indeed, inositol catabolism was significantly more abundant in the ruminal microbiome of the conventional group. Another enriched function in this group is the Lacto-N-Biose I Galacto-N-Biose Metabolic Pathway, which plays a role in the metabolism of mucin, a glycoprotein that is a component of mucus, which forms a protective physical barrier in the mucosal epithelium (Turroni et al., 2010). *Faecalibacterium prausnitzii* possesses this metabolic pathway (Blanco et al., 2019) and in addition to this species being identified in the metagenomes, its genus was found to be significantly enriched in the conventional group.

The complex carbohydrates contained in both diets, such as cellulose, hemicellulose and starch, are degraded to hexoses and pentoses (Ungerfeld, 2020). Hexoses can be converted to a complex and variable mixture of acids via the mixed acid fermentation pathway (Clark, 1989), another function enriched in the microbiome of the conventional group. Pentose-containing carbohydrates from the diet can be converted to glycolytic/gluconeogenic intermediates via the pentose phosphate pathway. This pathway is also involved in the metabolism of deoxyribose, deoxynucleoside, and purine (Price et al., 2019; Ungerfeld, 2020), whose catabolism and utilization were enriched in the microbiome of the conventional group.

In addition to carbohydrates, other key components in both diets are proteins and amino acids. The diet based on by-products likely increased the demand for protein degradation by the microbiomes since this function was identified as differentially abundant in the microbiome of the animals fed this diet. Dietary protein is broken down into amino acids and utilized by ruminal microorganisms to synthesize microbial protein, which is digested primarily in the small intestine (Lu et al., 2019). Soybean meal, which is included in the conventional diet, is rich in protein and high in lysine, an amino acid that is often lacking in cereal grains (Tian et al., 2019). In fact, the conventional diet group had a significantly higher abundance of functions related to lysine fermentation.

Regarding vitamins, cobalamin synthesis was found to be significantly enriched in the microbiome of the conventional group, whereas biotin synthesis and utilization in the microbiome of the by-products group. Cobalamin (also called vitamin B12) can be *de novo* synthesized by prokaryotes, such as *Pseudomonas dentrificans* (Fang et al., 2017). Biotin (also called vitamin H or B7) is an enzyme cofactor whose synthesis pathway is well characterized in *Escherichia coli* and *Bacillus subtilis* (Satiaputra et al., 2016). Accordingly, the genus *Pseudomonas* was significantly enriched in the microbiome of the conventional group, while the genera *Escherichia* and *Bacillus* were significantly enriched in the microbiome of the by-products group.

Urea in the diet transforms into microbial protein according to the fermentation rate of carbohydrates (Ding et al., 2015). As with the digestion of soybeans, its breakdown produces ammonia but also carbon dioxide (Hailemariam et al., 2021). In the microbiomes of the animals fed the conventional diet, which contained soybean meal along with more urea than the by-product diet, the function of ammonia assimilation was more abundant.

The by-product diet had more fat than the conventional diet, and this difference also impacted the microbiomes’ microbial functionality. Lipids are naturally found in the main components of both diets (corn, soybean and peanuts; Tessema et al., 2017). However, in the by-products diets, corn germ oil meal, which harbors the central part of the lipids of corn (Nascimento et al., 2022), was specifically added. The corn lipidic fraction is one of the main dietary sources of sphingolipids, such as galactosylceramide and sulfatide (Vesper et al., 1999; Sugawara et al., 2003). Functions related to the metabolism of these components were enriched in the fecal microbiome of the by-products group.

### 4.3 Associations between archaea, diet, and methane emission

In the microbiomes of animals fed the by-products diet, we observed a significantly higher diversity and abundance of archaea. However, the amount of methane emitted between the dietary groups was not significantly different. This was not surprising because although archaea play a primary role in methanogenesis, it has been reported that the abundance or diversity of methanogens does not reflect the amount of enteric methane emissions (Morgavi et al., 2012; Carberry et al., 2014). Thus, differences in methane emission could be more related to differences in the expression levels of genes that are part of methanogenic pathways, while it could also be related to the difference in the substrate supply to the methanogenic archaea (Shi et al., 2014; Tapio et al., 2017).

Indeed, we identified different archaeal genera that were enriched in each dietary group. The microbiomes of the conventional group had an enrichment of *Methanobrevibacter*, while, besides a high abundance of *Methanobrevibacter,* the microbiomes of the by-products group also had a significant enrichment in *Methanosphaera*. Both genera have been identified as dominant methanogenic archaea in the rumen, regardless of the geographical location or diet of the animals studied (Beauchemin et al., 2020). In our study, the effect of diet on the relative abundance of these genera is most likely due to their substrate specificity preference, as *Methanobrevibacter* is a hydrogenotrophic methanogen while *Methanosphaera* is a methylotrophic methanogen (Huws et al., 2018). This way, *Methanosphaera* would likely benefit from the methanol released during the fermentation of pectin (Mohnen, 2008) in the by-products diet.

Moreover, despite significant differences in the composition and functional potential of the GIT samples studied, we observed that the occurrence of methanogenic archaea remained the same regardless of the GIT sample studied. This finding reinforces what our group previously reported (Andrade et al., 2020), that fecal methanogens could be used as a proxy for rumen methanogens.

### 4.4 Associations between microbial diversity and cattle phenotypes

Our results demonstrated that microbial diversity was higher in fecal microbiomes of feed-efficient animals (assessed by RFI) fed the conventional diet and in fecal microbiomes of low-methane emitters (assessed by RME) fed the by-products diet. Thus, our results evidence the need for accounting on nutritional management when deriving phenotype associations.

Previous studies have associated a lower bacterial diversity with efficient ruminants (Guan et al., 2008; Shabat et al., 2016). However, they mainly focused on the ruminal microbiome, as the fecal microbiome only recently started to be evaluated in this regard. In agreement with our findings, a study on Angus steers with divergent feed efficiencies showed that the fecal microbial diversity was consistently higher in the most efficient steers during their productive lives (Welch et al., 2021). Another study investigated the relationship between the rumen, cecum, and fecal microbiomes and feed efficiency in finishing beef cattle. They observed that the Shannon diversity index values were greater in the cecum and feces of the efficient compared with the less efficient steers (Welch et al., 2020). The fecal microbiome mainly represents the microbiome present in the large intestine, which is where the breakdown of nutrients that escape the microbial digestion in the rumen and small intestine occurs. Therefore, a higher diversity of microorganisms in the large intestine could provide a greater array of enzymes capable of converting non-digested nutrients into metabolic end products that the host can utilize. This enhanced nutrient utilization could be a beneficial factor in efficient animals (Welch et al., 2020, 2021; Monteiro et al., 2022).

Regarding methane emission, a study comparing networks of coabundances from the microbiomes of high and low methane emitters found that the methanogenesis cluster in high methane emitters was dominated by a lower number of hydrogenotrophic methanogens with limited interaction, whereas the methanogenesis cluster in low methane emitters had more diverse methanogens involved in different methanogenic pathways and interacting more with other communities (Martínez-Álvaro et al., 2020). This is consistent with the results obtained here, where we observed a relationship between lower methane emissions and higher microbial diversity, and also with the by-products diet. The animals fed this diet had a higher diversity of archaea compared with the animals fed the conventional diet. A greater diversity of microorganisms in the microbiome may represent a greater number of microbes competing with methanogenic archaea by utilizing H_2_ or being involved in metabolic pathways known to have different effects on methane emissions, such as those related to amino acids, lactic acid or volatile fatty acids (VFA; Moss et al., 2000; Greening et al., 2019; Martínez-Álvaro et al., 2020).

### 4.5 Microbial genera and their potential functions linked to residual feed intake

Animals with low or negative RFI values are considered more efficient for consuming less feed than animals with high RFI, as would be expected for their body weight and tissue growth (Shabat et al., 2016). Thus, feed efficiency in beef cattle production is characterized by the ability of the GIT microbiome to convert potentially digestible feeds into metabolizable nutrients and provide energy to the host (Nkrumah et al., 2006). Therefore, it is coherent that animals with higher feed efficiency harbor more microbes that perform functions related to nutrient metabolism and energy utilization (Xue et al., 2022). And indeed, in our study, when the functional potentials of ruminal taxa associated with efficient cattle and inefficient cattle were compared, a greater abundance of functions related to the metabolism of monosaccharides, di/oligosaccharides, and amino sugars was found in the former. And in fact, several of these taxa are known to be xylanolytic and cellulolytic bacteria, such as *Cellulomonas*, *Sorangium, Actinomadura, Actinoplanes, Rubrobacter*, and *Burkholderia* (Schneiker et al., 2007; Saini et al., 2015). In addition, *Succinivibrio* and *Brevibacterium* have been previously associated with efficient cattle, in agreement with our study (McCormack et al., 2017; Elolimy et al., 2020; Zhang et al., 2021).

In contrast, among the four taxa significantly associated with inefficient cattle, at least two hardly use carbohydrates for growth. *Acidaminococcus* uses amino acids as its sole source of energy for growth (Rogosa, 1969). Diverse *Phascolarctobacterium* species are asaccharolytic and use succinate as a substrate (Watanabe et al., 2012; Ikeyama et al., 2020). *Selenomonas* is not a complex polysaccharide degrader, it utilizes soluble carbohydrates released by other ruminal bacteria (Ricke et al., 1996).

Consistent with our study, these genera or species within these genera have previously been associated with feed-inefficient ruminants (Hernandez-Sanabria et al., 2012; Shabat et al., 2016; Auffret et al., 2020; Costa-Roura et al., 2021; Zhang et al., 2021; Xie et al., 2022). However, some of them have also been associated with feed-efficient ruminants (Auffret et al., 2020; Elolimy et al., 2020; Xue et al., 2022). These disparities could be attributed to variations in the functional roles of these microbial taxa within different animal hosts and studies.

Differences in functions related to protein metabolism were also observed among taxa that were significantly associated with feed efficiency, such as the higher abundance of protein biosynthesis in taxa associated with inefficient cattle. Since nutrient degradation is an essential step that characterizes efficient cattle, higher abundances of protein biosynthesis functions in the taxa associated with feed inefficiency could represent a delay or deviation of resources. It has been suggested that inefficient animals use resource compounds more diversely than efficient ones, which could result in the production of metabolites that will not be utilized by the animal for its energy requirements or with the potential to negatively affect the animal’s energy harvest (Shabat et al., 2016). Meanwhile, we observed in our study that taxa related to efficient cattle had higher abundances of protein folding and degradation. The microbiomes of efficient animals appear to utilize the relevant and valuable compounds in metabolic pathways more related to their energy needs (Shabat et al., 2016).

It should be noted that although these taxa and their putative functions could be associated with the phenotype index, the association does not imply causality, and the presence of genes identified based on metagenomic data does not guarantee that they are being expressed and functional. Further studies using metatranscriptomics and proteomics may shed light on the hypotheses raised here.

### 4.6 Microbial genera and their potential functions linked to residual methane emission

In our study, several ruminal and fecal bacteria were significantly associated with high methane emission, as well as two archaea. Methane can be synthesized following three main pathways: hydrogenotrophy, methylotrophy, and acetoclastic methanogenesis (Martínez-Álvaro et al., 2020). The substrates required for these processes (H_2_, formate, methyl compounds, ethanol, and acetate) are produced by bacteria fermenting dietary and microbial components (Janssen and Kirs, 2008). In this way, bacterial populations and also fungi and protists that are part of the GIT microbiomes interact with methanogens and influence methane emissions (Martínez-Álvaro et al., 2020).

*Methanococcus*, one of the archaea we found to be significantly associated with high methane emission, is a hydrogenotrophic cytochrome-lacking methanogen that uses H_2_/CO_2_ or formate to produce methane (Sparling and Daniels, 1986). By checking the functions attributed to the reads classified as *Methanococcus,* we identified genes that encode methanofuran, tetrahydromethanopterin, coenzyme F420, coenzyme B, and coenzyme M, which are coenzymes and cofactors known to be used by hydrogenotrophic archaea as carbon and electron carriers during methanogenesis (Richards et al., 2016).

Bacteria associated with high methane emission in our study as well as *Thermococcus*, are not methane producers; however, some of their reads were classified as genes that encode some specific metalloenzymes ([NiFe]- and [FeFe]-hydrogenases) and isoenzymes (formate hydrogenases and dehydrogenases). These enzymes are part of the fermentation pathways that lead to H_2_ catalysis and formate production/consumption (Hungate et al., 1970; Greening et al., 2019). This may influence methane production/emission since H_2_ is the main substrate used for methanogenesis, and formate is an important electron donor that has been estimated to account for up to 18% of the methane produced in the rumen (Hungate et al., 1970; Tapio et al., 2017). The main taxa that we identified in association with increased methane emission (*Pseudobutyrivibrio*, *Anaerobutyricum,* and *Roseburia*) had a higher abundance of these hydrogenases. These genera, as well as most other genera that were associated with high methane emission, belong to the well-characterized butyrate-producing *Lachnospiraceae* family (Vital et al., 2014; Martínez-Álvaro et al., 2020; Smith et al., 2022a). The formation of this short-chain fatty acid (SCFA) may result in the production of formate and H_2_ (Moss et al., 2000). Therefore, our results suggest that those taxa influence methane production by contributing the substrates needed for methanogenesis. These substrates could benefit not only *Methanococcus* but also *Methanobrevibacter*, which is the most abundant archaeal genus identified in the metagenomes and also a hydrogenotrophic methanogen (Huws et al., 2018). Interestingly, these three genera (*Pseudobutyrivibrio*, *Anaerobutyricum*, and *Roseburia*) were also significantly enriched in the conventional group, demonstrating once again the diet effect on the production phenotypes. Besides those, *Eubacterium*, *Novisyntrophococcus*, *Lacrimispora*, *Mediterraneibacter*, *Coprococcus*, and *Anaerostipes* were significantly associated with both conventional diet and high methane emission. No genus significantly associated with the by-products diet were associated with the phenotypes indexes.

Moreover, the *Eubacterium* genus was associated with both high-RFI (inefficient animals) and high-RME (high methane emitters). This is in line with the concept that methane production in rumen is negatively associated with host feed efficiency (Nkrumah et al., 2006; Zhou et al., 2009; Carberry et al., 2014). However, it is still not clear whether methane emission variations are directly related to feed efficiency or the fact that efficient animals have lower DMI, and, therefore, less amount of substrates available for fermentation and hydrogen formation (Sakamoto et al., 2021). Inversely, *Winogradskyella* was associated with a decrease in methane emission. Although its family, *Flavobacteriaceae*, has been found in animal digestive tracts, this particular genus has so far been exclusively found in marine environments (Thoetkiattikul et al., 2013; Alejandre-Colomo et al., 2021). Given its distant origin relative to the rumen microbiome, the identification of a marine bacteria (and other microorganisms) in the rumen microbiome raises the possibility that this is a misclassification related to database biases. The rumen microbiome is still an underexplored environment, and therefore, metagenomic sequences may be misclassified or unclassified when derived from novel or uncultured microbes that are not present or well-represented in reference databases (Smith et al., 2022b). More comprehensive searches are imperative to validate these identifications.

Interestingly, acetogens such as *Blautia*, *Eubacterium*, and *Sporomusa*, known to produce acetate from H_2_ and CO_2_ (Karekar et al., 2022), were also associated with high methane emission. By consuming H_2_, these bacteria redirect its uptake away from methanogenesis and based on that, their stimulation has been previously proposed as a strategy for methane mitigation in methanogen-inhibited scenarios (Choudhury et al., 2022). However, this idea has some conflicting points considering that as ruminal acetogens can utilize a wide range of organic substrates, they can even contribute to H_2_ production and cannot compete for H_2_ with methanogens under normal circumstances (Lopez et al., 1999; Choudhury et al., 2022; Kelly et al., 2022). This hypothesis may hold true for the microbiomes in this study, but further analyses are needed.

## Data availability statement

The data presented in the study are deposited in the National Center for Biotechnology Information repository, accession number PRJNA987743.

## Ethics statement

The animal study was approved by EMBRAPA Livestock Science Ethics Committee on Animal Experimentation, São Carlos, São Paulo (Protocol No. 09/2016). Experimental procedures were conducted following Brazilian guidelines on animal welfare and approved by the Ethics Committee on the Use of Animals, College of Veterinary and Animal Science, São Paulo State University under protocol n° 8510190118. The study was conducted in accordance with the local legislation and institutional requirements.

## Author contributions

LiC: Conceptualization, Formal Analysis, Methodology, Writing – original draft, Investigation, Validation, Visualization. JS: Writing – original draft, Investigation. BA: Conceptualization, Writing – review & editing, Investigation. TC: Writing – review & editing, Investigation. JB: Visualization, Writing – review & editing, Investigation. PO: Writing – review & editing, Investigation. GM: Supervision, Writing – review & editing, Data curation, Funding acquisition, Project administration. LuC: Supervision, Writing – review & editing, Funding acquisition, Project administration. JP: Supervision, Writing – review & editing, Data curation. AB: Supervision, Writing – review & editing, Data curation. SM: Supervision, Writing – review & editing, Data curation. LR: Conceptualization, Funding acquisition, Project administration, Resources, Supervision, Writing – review & editing.

## Footnotes

^1^ http://pubseed.theseed.org/
