# Supplementary material for: Rumen and fecal microbiomes are related to diet and production traits in Bos indicus beef cattle
Source: Front Microbiol. 2023 Dec 15;14:1282851. doi: 10.3389/fmicb.2023.1282851 (PMC10754987; doi:10.3389/fmicb.2023.1282851)
Supplement: Supplementary file 10 [file Image_1.pdf]

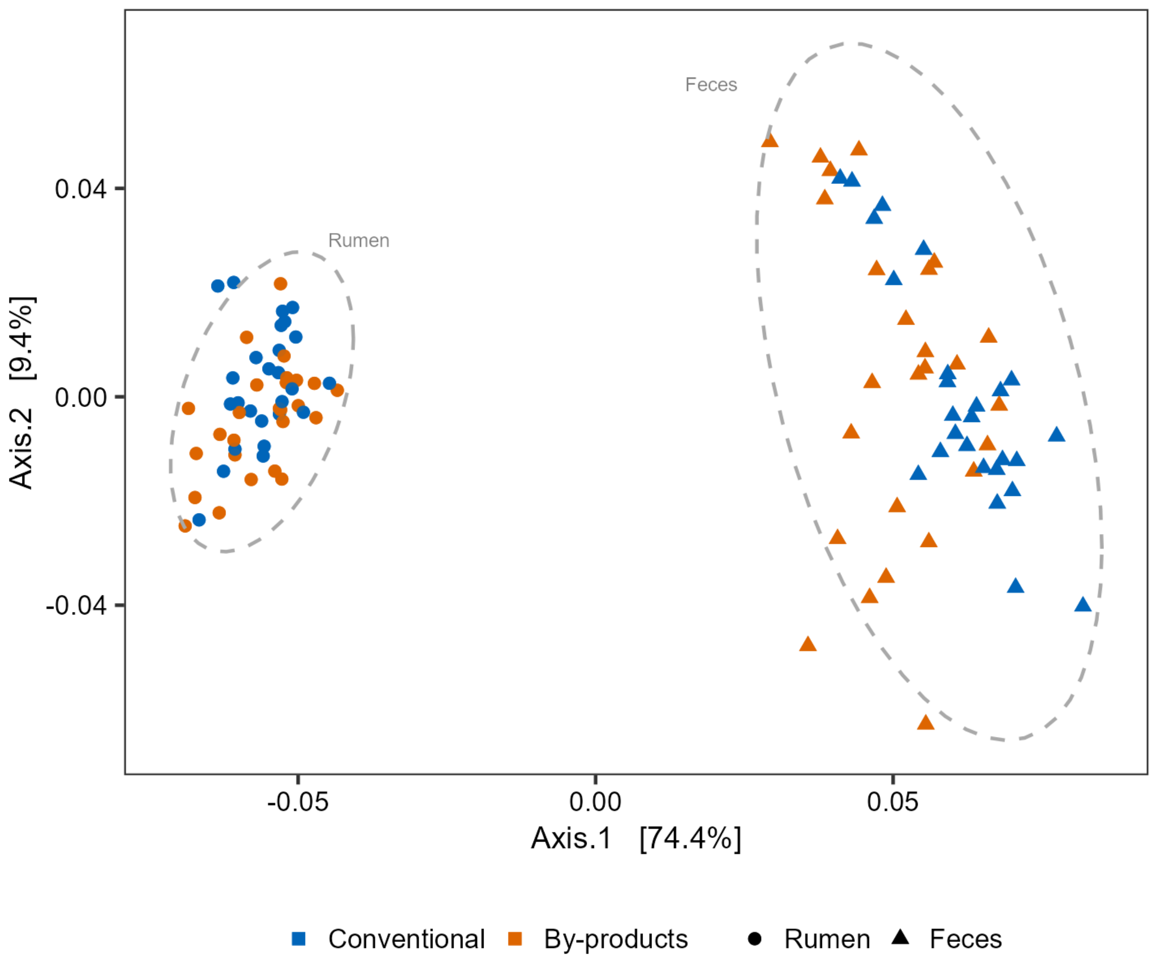

Supplementary Figure 1. Beta-diversity analysis based on the microbiome's functions. Principal coordinate analysis (PcoA) generated with Bray–Curtis dissimilarity distances of functions at level 3 identified in the metagenomes
