# Supplementary material for: Rumen and fecal microbiomes are related to diet and production traits in Bos indicus beef cattle
Source: Front Microbiol. 2023 Dec 15;14:1282851. doi: 10.3389/fmicb.2023.1282851 (PMC10754987; doi:10.3389/fmicb.2023.1282851)
Supplement: Supplementary file 11 [file Image_2.pdf]

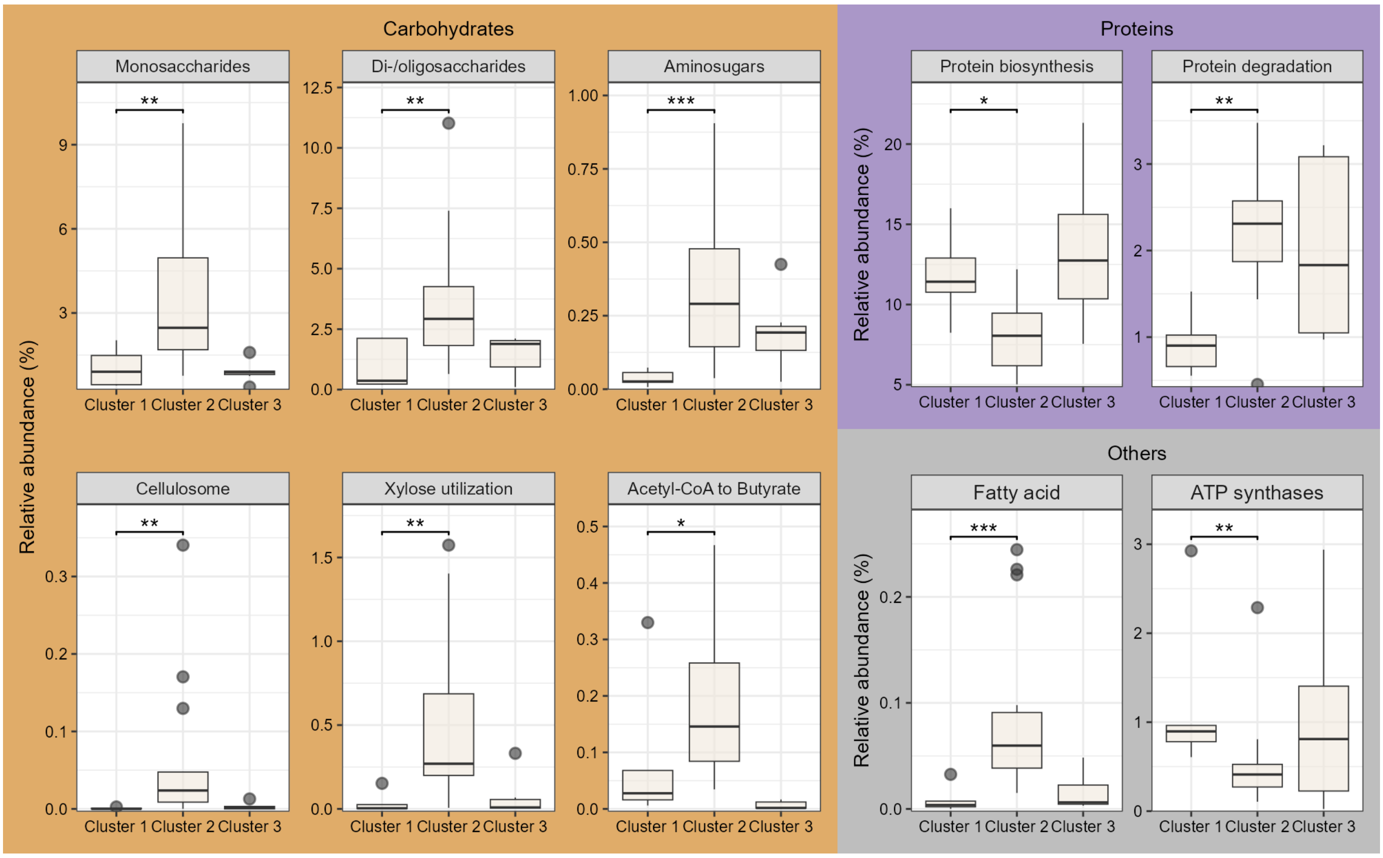


**Supplementary Figure 2.** Functional differences between the clusters associated with RFI. Boxplots showing the abundance (percentage) of level 2 and 3 functions, considering the clusters of taxa significantly associated with RFI. * P <= 0.05, ** P <= 0.01, ***P < 0.001 (Wilcoxon test)
