# Supplementary material for: Rumen and fecal microbiomes are related to diet and production traits in Bos indicus beef cattle
Source: Front Microbiol. 2023 Dec 15;14:1282851. doi: 10.3389/fmicb.2023.1282851 (PMC10754987; doi:10.3389/fmicb.2023.1282851)
Supplement: Supplementary file 12 [file Image_3.pdf]

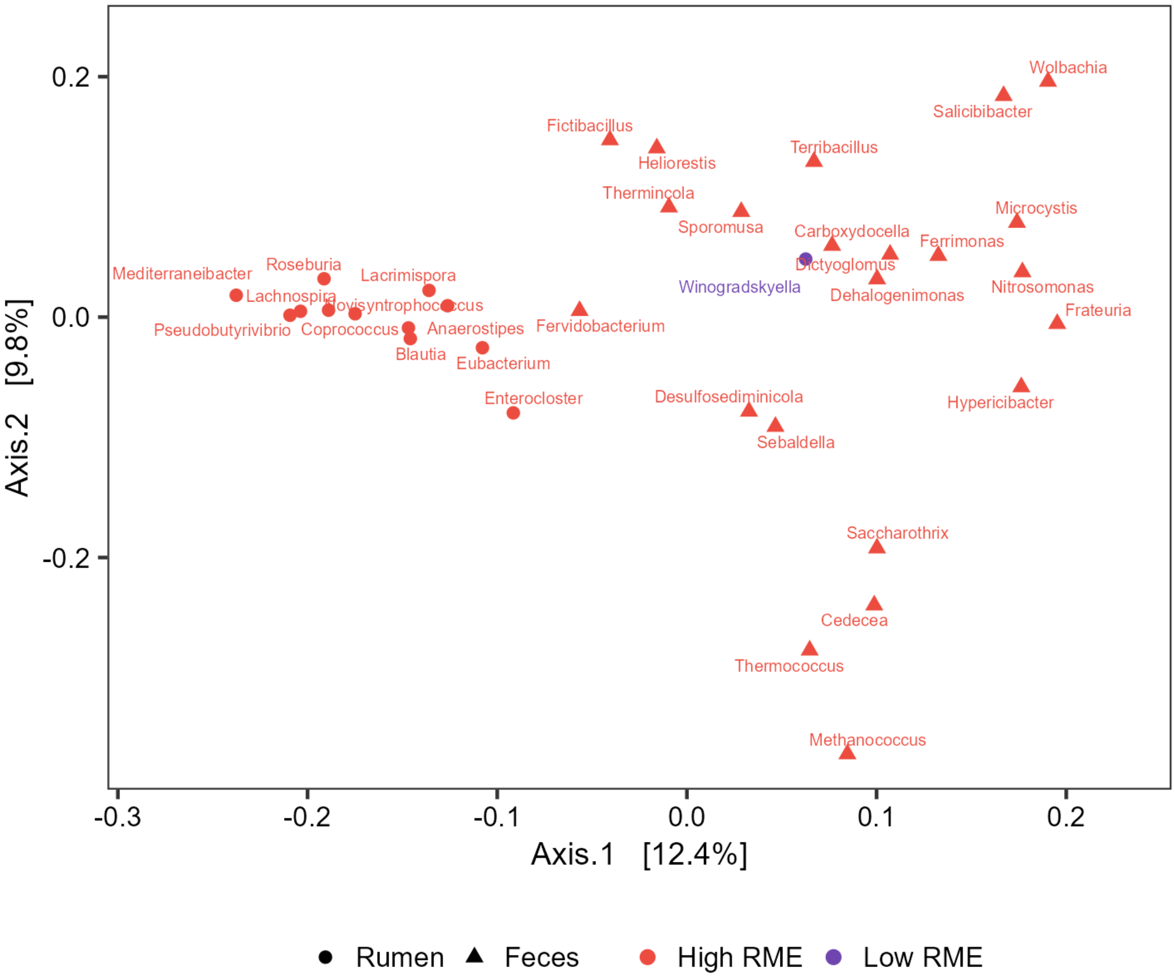

Supplementary Figure 3. Beta-diversity analysis based on the functions related to the RME-associated taxa. Principal coordinate analysis (PcoA) generated with Bray–Curtis dissimilarity based on the functional profile of each taxon significantly associated with RME
